# Refractory atrial arrhythmias in Duchenne muscular dystrophy: a case series

**DOI:** 10.1016/j.hrcr.2024.07.023

**Published:** 2024-08-02

**Authors:** Eleanor Greiner, Chet R. Villa, Thomas D. Ryan, David S. Spar

**Affiliations:** 1 Cincinnati Children’s Hospital Medical Center, Cincinnati, Ohio

**Keywords:** Duchenne muscular dystrophy, Atrial arrhythmias, Cardiomyopathy, Fibrosis, Catheter ablation

**Key Teaching Points**

• Patients with DMD are at risk for atrial arrhythmias.
• The arrhythmias in DMD patients can be difficult to control with both medication and invasive electrophysiology procedures.
• Atrial fibrillation can contribute to the morbidity and mortality of patients with DMD.

## Introduction

Duchenne muscular dystrophy (DMD) is an X-linked disorder that results from mutations in the dystrophin gene; it is characterized by fibrofatty replacement of skeletal and cardiac muscle. Recent studies have demonstrated that both the atrial and ventricular myocardium are affected in DMD, placing these patients at risk for not only dysfunction, but also for the development of ventricular and atrial arrhythmias.^1^ In addition to structural myocardial changes, patients with DMD often have comorbidities that further increase their risk of arrhythmias, such as sleep disordered breathing and increased adiposity from chronic steroid use.^2^^,^^3^

While the risk of atrial arrhythmias has been recognized in DMD patients, to date there is no standard approach to management.^4^ DMD is rare, and patients are often excluded from trials looking at the benefits and risks of antiarrhythmic therapy and catheter ablation.^5^ The most recent expert consensus statement on the evaluation and management of arrhythmias in neuromuscular disorders comments on the risk of atrial arrhythmias in DMD patients, but the only management discussed is anticoagulation.^5^

The current case series presents 5 patients with DMD, all of whom developed atrial arrhythmias in their teenage to early adult years which proved to be difficult to manage. This series highlights the propensity for atrial arrhythmias in DMD patients as they age, and reviews both medical and interventional therapies used.

## Case reports

Patient 1 developed symptomatic atrial fibrillation with rapid ventricular response (RVR) at age 13 (Supplemental Figure 1). On initial presentation, he was found to have an appreciable change in function (left ventricular ejection fraction [LVEF] 17% from baseline in mid-30s). He was not receiving supplemental respiratory support at the time, and his forced vital capacity (FVC) was 115% predicted. He started amiodarone in addition to maximally tolerated guideline-directed medical therapy. With control of his arrhythmia, his LVEF improved to approximately 40% on echo before hospital discharge. Subsequent imaging has shown LVEF of 30%–35% over the long term. After no breakthrough episodes for over 1 year, amiodarone was discontinued to mitigate long-term toxicity risks. Atrial fibrillation recurred within 4 months; therefore, the patient received a trial of flecainide. He developed multiple breakthrough episodes of symptomatic atrial fibrillation despite flecainide, requiring re-initiation of amiodarone within 6 months and a dose increase shortly thereafter. He then underwent electrophysiology study (EPS) and ablation at age 16. In the laboratory, he had no other mechanism of supraventricular tachycardia, with no dual AV nodal physiology and no concealed accessory pathways or ectopic atrial tachycardia foci. His atrial fibrillation was inducible only with large adenosine boluses. He underwent pulmonary vein isolation with a cryoballoon. Unfortunately, he had recurrence of atrial fibrillation within 4 months; thus, amiodarone was restarted. His atrial fibrillation proved to be well controlled with anti-arrhythmic medication; however, he developed amiodarone-associated thyroid disease requiring discontinuation. After discontinuation, he had recurrent arrhythmia episodes, and a subsequent trial with dofetilide resulted in a prolonged QTC (>500 ms) and recurrent atrial fibrillation with RVR. He went back to using amiodarone with modification of his existing steroid therapy, which has been maintained since that time. At present, he has rare breakthrough episodes of atrial fibrillation and requires ongoing treatment of his thyroid disease (Table 1).

**Table 1:** Clinical patient data

Patient	Age at onset (years)	Baseline ECG	Arrhythmia type	Left ventricular ejection fraction at diagnosis	LGE at diagnosis	Left atrial volume at diagnosis	Anti-arrhythmic medications trialed	Invasive EP interventions and results	Outcome
1	13	Sinus rhythm	- NSVT - Afib with RVR	17%	Yes; basal anterolateral, anteroseptal, inferolateral; mid-ventricular anterolateral, anteroseptal inferolateral, apex	Normal	Amiodarone, metoprolol, flecainide, dofetilide	- Pulmonary vein isolation with cryoballoon - Afib Recurrence within 3 months	Living; rare breakthrough episodes of Afib while on amiodarone
2	19	Sinus rhythm, LAD, nonspecific intraventricular conduction delay	Atrial tachycardia	20%	Yes; basal anterior, anteroseptal, inferior inferolateral, anterolateral; mid anterior, inferolateral and anterolateral; apical anterior and lateral	Normal	Amiodarone, metoprolol	- ICD placement - AV node ablation - Recurrence within 1 month	Deceased; recurrent atrial tachycardia, progressive heart failure, multiorgan failure
3	17	Sinus rhythm, biventricular hypertrophy	- NSVT - Afib	35%	Yes; basal anterolateral, inferolateral; mid-anterolateral, inferolateral, inferoseptal; apical anterior, lateral, inferior	Normal	Metoprolol, amiodarone	None	Living; well controlled with amiodarone
4	19	Sinus rhythm, LAE, right ventricular hypertrophy	- Afib - Atrial flutter	Moderate dysfunction (qualitative)	Unknown (no MRI with contrast prior to onset of arrhythmia)	Normal	Amiodarone, metoprolol	- Cavotricuspid isthmus (CTI) radiofrequency ablation - No known recurrence of atrial flutter	Living; breakthrough episodes of Afib
5	24	Sinus rhythm, LAE, LAD, nonspecific intraventricular conduction delay	- Atrial tachycardia - NSVT	20%	Yes; basal and mid-ventricular anteroseptal, inferoseptal, anterior, anterolateral, inferolateral inferior; apical lateral	Normal	Amiodarone	None	Deceased; cardiac arrest at home with ROSC following defibrillation; UGI bleed with hemorrhagic shock during post-arrest admission

Afib = atrial fibrillation; ECG = electrocardiogram; EP = electrophysiologic; ICD = implantable cardioverter defibrillator; LGE = late gadolinium enhancement; LAD = left axis deviation; LAE = left atrial enlargement; NSVT = non-sustained ventricular tachycardia; ROSC = return of spontaneous circulation; RVR = rapid ventricular response; UGI = upper gastrointestinal.

**Figure 1:**
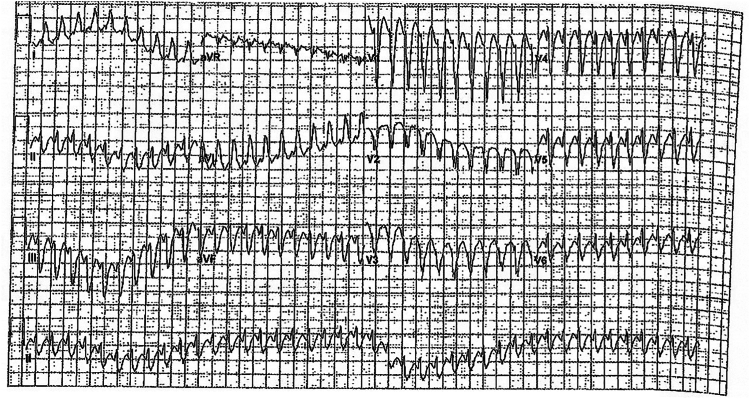
Electrocardiogram from Patient 2 on initial presentation, found to be in a wide complex tachycardia with a superior axis and an interventricular conduction delay.

Patient 2 was 19 years old when he developed wide complex tachycardia with a rate of 250 beats per minute (bpm). This was thought to be SVT conducted with aberrancy (Figure 1; Supplemental Figure 2). This was in the setting of severe ventricular dysfunction (LVEF 20%) and poor respiratory status (FVC 33% predicted) requiring nightly Bilevel positive airway pressure (BiPAP). A few days later, he underwent an EPS which revealed dual AV nodal conduction as well as inducible, sustained typical AV nodal reentry tachycardia which ultimately degenerated into atrial fibrillation. He also had an interventricular conduction delay with a left bundle-branch block-type pattern. He underwent radiofrequency ablation as well as cardiac resynchronization therapy defibrillator placement for primary prevention. He was ultimately discharged on amiodarone and metoprolol. He subsequently had multiple episodes of atrial tachycardia, mostly conducted 2:1, but occasionally with 1:1 conduction. In this setting he received multiple inappropriate shocks for rapidly conducting atrial tachycardia. His metoprolol succinate was up titrated to maximally tolerated doses and atrial anti-tachycardia pacing was added to his therapies. His arrhythmia briefly subsided; however, between 12 and 18 months after presentation, he was admitted 3 times for recurrent atrial tachycardia with rapid conduction and with intermittent inappropriate shocks. Owing to recurrent arrhythmia, he was presented with an option of atrial tachycardia ablation with possible AV node ablation if multiple foci were identified. The latter intervention was offered to avoid prolonged intubation in the setting of severe lung disease (FVC 35% predicted). He ultimately underwent a repeated EPS at age 21, and it demonstrated multiple atrial tachycardias with at least 3 different cycle lengths. Because of the preoperative considerations, the presence of multiple foci and the patient’s wishes, he underwent AV node ablation rather than a prolonged EPS to address the multiple foci. Within a month of the procedure, he developed recurrent atrial tachycardia. He had an atrial rate of 170 ms with AV block with a biventricular paced rhythm of 750 ms noted on device interrogation. This episode was associated with progressive heart failure symptoms requiring admission to the cardiac intensive care unit. His condition continued to deteriorate throughout his admission, and he ultimately experienced multiorgan dysfunction. He died at age 21 (about 2 years from initial atrial tachycardia presentation) owing to decompensated heart failure (Table 1).

Patient 3 was first noted to have episodes of asymptomatic, nonsustained ventricular tachycardia (NSVT) with rates of 142–158 bpm on routine Holter monitoring at age 17–18 (Supplemental Figure 3). At the time, his LVEF was moderately diminished (approximately 35%) with evidence of late gadolinium enhancement. He was not receiving ventilator support at the time, and his FVC was 91% predicted. Given episodes of NSVT in the setting of significant left ventricular systolic dysfunction, discussions were had with the patient about implantable cardioverter defibrillator placement. To inform the decision, an implantable rhythm monitor was placed (age 19) to understand the true ventricular tachycardia burden and to monitor for atrial arrhythmias, which if present would put him at risk for inappropriate shocks. Two months into monitoring, he was noted to have an episode of atrial fibrillation. He had his second episode of atrial fibrillation with RVR (with rates of 200–220 bpm) about 3 months later, at which time his carvedilol was switched to metoprolol succinate and his dose was optimized. Over the next year the patient had recurrent episodes of atrial fibrillation with RVR, prompting an admission for amiodarone initiation. In the past year since starting daily amiodarone therapy, his arrhythmia burden has been well controlled with no further episodes of atrial fibrillation (Table 1).

Patient 4 came to the cardiomyopathy clinic at age 19 and was found to be hypotensive in the setting of new onset atrial fibrillation (Supplemental Figure 4). Echocardiography at the time revealed moderate global left ventricular dysfunction (visual estimate of LVEF approximately 40% owing to poor acoustic windows), which had worsened from 2 years prior. He was using nightly BiPAP for respiratory support with an FVC 27% predicted. He underwent cardioversion and started warfarin. Seven months later, he was readmitted for a similar presentation requiring repeat cardioversion. Two weeks later, he had his third admission for atrial fibrillation requiring cardioversion, prompting initiation of amiodarone. On this therapy, he remained arrhythmia free for approximately 4 years with improvement in LV systolic function (mildly diminished with LVEF approximately 50%). At age 25, the patient was admitted for recurrent atrial fibrillation with RVR, but he spontaneously converted to sinus rhythm before cardioversion. He was noted to have intermittent, short-lived episodes of atrial fibrillation on monitoring and during hospitalizations (respiratory infections). At age 31, the patient developed recurrent episodes of atrial flutter (documented on ECGs) in addition to more frequent episodes of symptomatic atrial fibrillation, which were unable to be controlled with amiodarone. Metoprolol succinate was added without significant benefit. As a result, he underwent cardiac catheterization with EPS. His catheterization revealed mildly elevated right and left ventricular filling pressures with low-normal cardiac output. EPS showed no inducible tachycardia and dual AV nodal physiology. Based on clinically recorded typical atrial flutter, cavotricuspid ablation was performed in an anatomical fashion. The procedure was well tolerated, and he has had no clinical recurrence of atrial flutter to date. He continues to have episodes of atrial fibrillation for which he continues to take amiodarone (Table 1). His last ambulatory rhythm monitor demonstrated 10% atrial fibrillation burden.

Patient 5 was first noted to have brief runs of asymptomatic atrial tachycardia on routine Holter monitoring at age 24 (Supplemental Figure 5). At that time, he had severe left ventricular and moderate right ventricular dysfunction with extensive late gadolinium enhancement. He was receiving appropriate heart failure therapies including sacubitril/valsartan, carvedilol, digoxin, and spironolactone. He used supplemental BiPAP at night but no respiratory support during the day. His FVC at the time was 18% predicted. For the next 2.5 years, the patient had short runs of atrial tachycardia (with rates in the 130s–190s bpm) and episodes of NSVT (with rates in the 130s–170s bpm) on yearly Holter and event monitor analysis. He had no episodes of symptomatic arrhythmias during this time. At age 27, the patient became acutely unresponsive at home, requiring 8 minutes of CPR. Upon arrival of EMS, he was found to be in a wide complex rhythm and received one 200-J shock with return of spontaneous circulation (ROSC). He underwent intubation and was taken to an outside hospital. Review of the EMS tracings from the event revealed a likely rapidly conducting atrial tachycardia which was aborted successfully with defibrillation. Echocardiography on admission showed worsening function from baseline (LVEF <10% from baseline LVEF of 20%–25%). He was also noted to be positive for *Haemophilus influenza* on a respiratory culture. During his initial days in the hospital, he had recurrent episodes of atrial tachycardia and atrial flutter requiring initiation of amiodarone. He eventually underwent extubation and was transferred to a tertiary care center for ongoing management. At the center, his home heart failure medications were started and amiodarone was continued, with resolution of atrial arrhythmias. The patient briefly used rivaroxaban for anticoagulation but transitioned to aspirin early in his hospital course. Unfortunately, he developed progressive heart failure and transitioned to comfort care. While in the hospital, he developed an acute upper gastrointestinal bleed with development of hemorrhagic shock, which ultimately led to his death (Table 1).

## Discussion

This case series presents data on 5 individuals with DMD who developed refractory atrial arrhythmias in their teenage to young adult years. Three of these patients underwent ablation owing to recurrent arrhythmias while receiving maximally tolerated medical therapy. Unfortunately, the procedure had limited durability in all 3 patients, with a return of atrial arrhythmias within days to a few months of the procedure. Generally, the 12-month recurrence rate of atrial fibrillation following ablation in the adult population is 20%–50%.^6^, ^7^, ^8^ While too small for statistical analysis, this series of patients suggests a higher failure rate than in the general population.

There are likely several factors that play into the difficulty of atrial arrhythmia treatment in patients with DMD, but one factor to consider is the presence of multiple arrhythmogenic foci in the atrium related to diffuse, patchy fibrofatty replacement of the atrium. Recent autopsy data suggest that the atria in patients with DMD is affected by pathologic fibrofatty infiltration in a similar manner as the ventricles. In advanced disease, the atrium becomes affected, initially in a patchy way, with progression to diffuse, full-thickness myocardial replacement.^1^ Fibrotic tissue disrupts healthy cardiomyocytes and can alter electrophysiological properties putting patients at risk for focal firing and re-entrant circuits.^3^ Thus, in patients with DMD, ablation may prove to be less successful because of the diffuse changes seen in the atrium as opposed to focal areas of disease. While not observed in our cohort, the risk of complications such as perforation during a catheter-based procedure may be higher in this population given the thin atrial walls in the setting of pathologic fatty replacement.

Atrial fibrillation is an important source of morbidity and mortality in the adult population, increasing the risk of stroke and heart failure symptoms. Of the patients included in this series, none experienced stroke or any thromboembolic complications, although most were treated with some form of anticoagulation. In terms of heart failure symptoms, most of the patients included were found to have a decreased EF at some point in their time course related to frequent arrhythmia. Patient 2 died from multiorgan dysfunction related to decompensated heart failure, which progressed relatively rapidly following the onset of atrial fibrillation 2 years before his death.

## Conclusion

The patients included in this case series demonstrate that atrial arrhythmias can be problematic in patients with DMD and can contribute to morbidity and mortality. However, there is not an easy solution, as most cases prove to be recalcitrant with medical management and unresponsive to ablations. This lack of solutions highlights the difficult balance between risk and benefit when it comes to treating arrhythmias in this patient population and the need for long-term, multicenter studies given the rarity of this disease.

## Disclosures

Chet R. Villa was previously a consultant for 10.13039/100013223PTC Therapeutics, 10.13039/100004319Pfizer and 10.13039/100014943Sarepta, and remains a current consultant for Capricor, Antisense, and Solid Biosciences; he has also received funding from the Parent Project Muscular Dystrophy; however, no funds were used in the creation of this work. All other authors have no conflicts to disclose.

## References

[1] Greiner E., Breaux A., Kasten J. (2024). Cardiac atrial pathology in Duchenne muscular dystrophy. Muscle Nerve.

[2] Hurvitz M.S., Sunkonkit K., Massicotte C., Li R., Bhattacharjee R., Amin R. (2022). Characterization of sleep-disordered breathing in children with Duchenne muscular dystrophy by the American Academy of Sleep Medicine criteria vs disease-specific criteria: what are the differences?. J Clin Sleep Med.

[3] Sagris M., Vardas E.P., Theofilis P., Antonopoulos A.S., Oikonomou E., Tousoulis D. (2021). Atrial fibrillation: pathogenesis, predisposing factors, and genetics. Int J Mol Sci.

[4] Villa C.R., Czosek R.J., Ahmed H. (2015). Ambulatory monitoring and arrhythmic outcomes in pediatric and adolescent patients with Duchenne muscular dystrophy. J Am Heart Assoc.

[5] Groh W.J., Bhakta D., Tomaselli G.F. (2022). 2022 HRS expert consensus statement on evaluation and management of arrhythmic risk in neuromuscular disorders. Heart Rhythm.

[6] Poole J.E., Bahnson T.D., Monahan K.H. (2020). Recurrence of atrial fibrillation after catheter ablation or antiarrhythmic drug therapy in the CABANA Trial. J Am Coll Cardiol.

[7] Calkins H., Hindricks G., Cappato R. (2017). 2017 HRS/EHRA/ECAS/APHRS/SOLAECE expert consensus statement on catheter and surgical ablation of atrial fibrillation: executive summary. Heart Rhythm.

[8] Chew D.S., Black-Maier E., Loring Z. (2020). Diagnosis-to-ablation time and recurrence of atrial fibrillation following catheter ablation: a systematic review and meta-analysis of observational studies. Circ Arrhythm Electrophysiol.

