# Supplementary material for: Refractory atrial arrhythmias in Duchenne muscular dystrophy: a case series
Source: HeartRhythm Case Rep. 2024 Aug 2;10(11):798–802. doi: 10.1016/j.hrcr.2024.07.023 (PMC11628784; doi:10.1016/j.hrcr.2024.07.023)
Supplement: Supplemental Figures S1–S5 [file mmc1.pptx]

## Slide 1
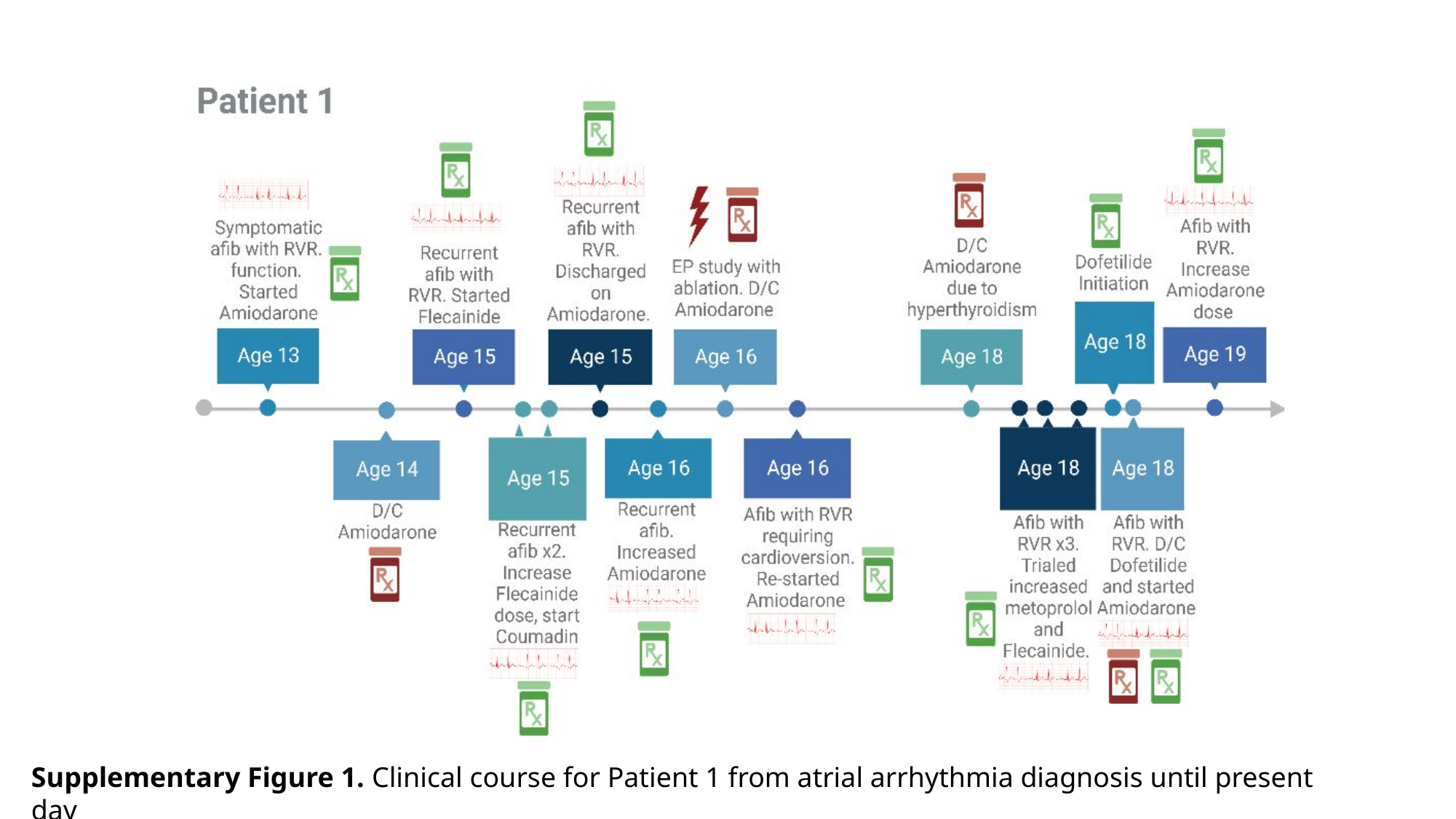

Supplementary Figure 1. Clinical course for Patient 1 from atrial arrhythmia diagnosis until present day

## Slide 2
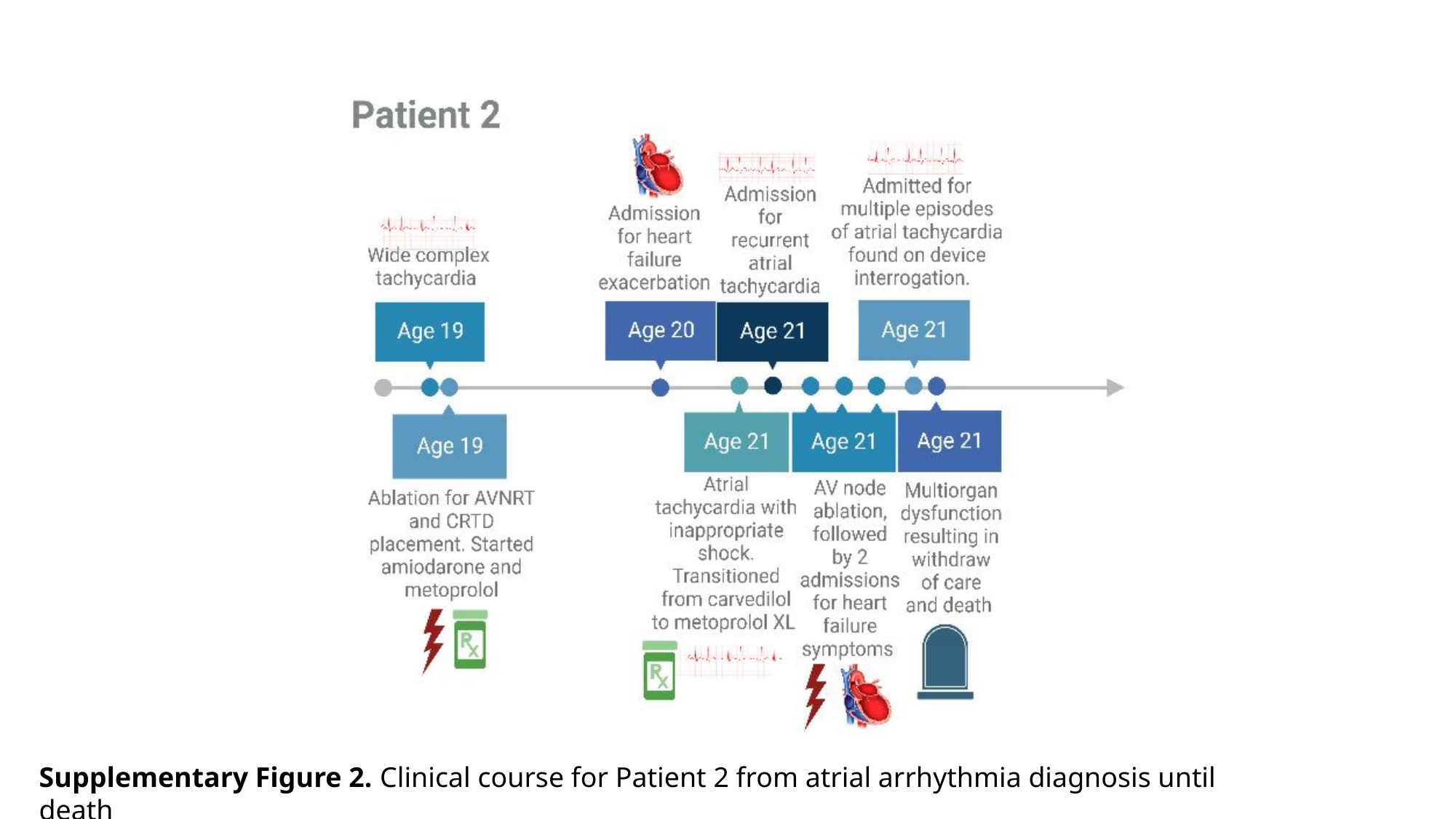

Supplementary Figure 2. Clinical course for Patient 2 from atrial arrhythmia diagnosis until death

## Slide 3
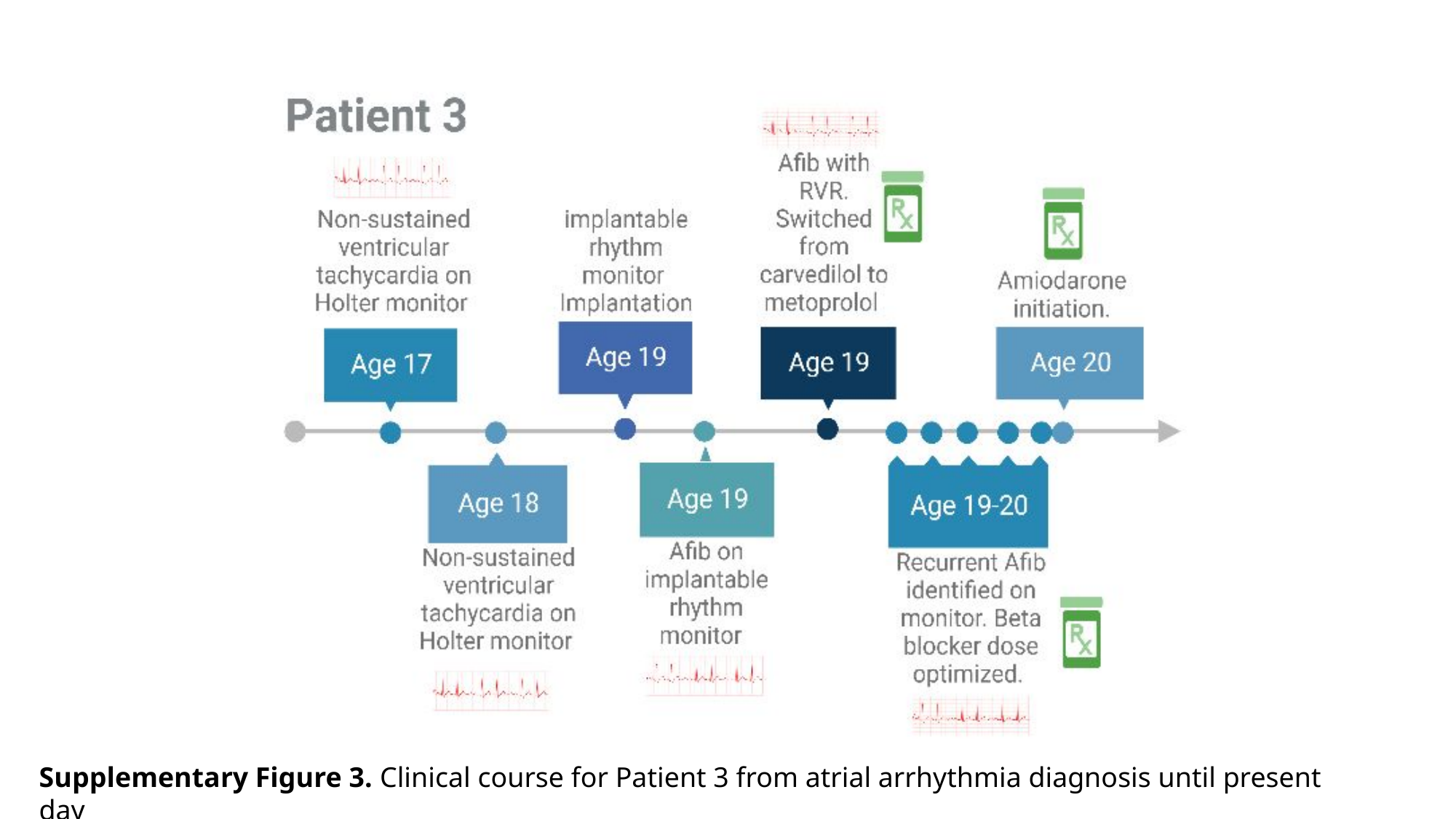

Supplementary Figure 3. Clinical course for Patient 3 from atrial arrhythmia diagnosis until present day

## Slide 4
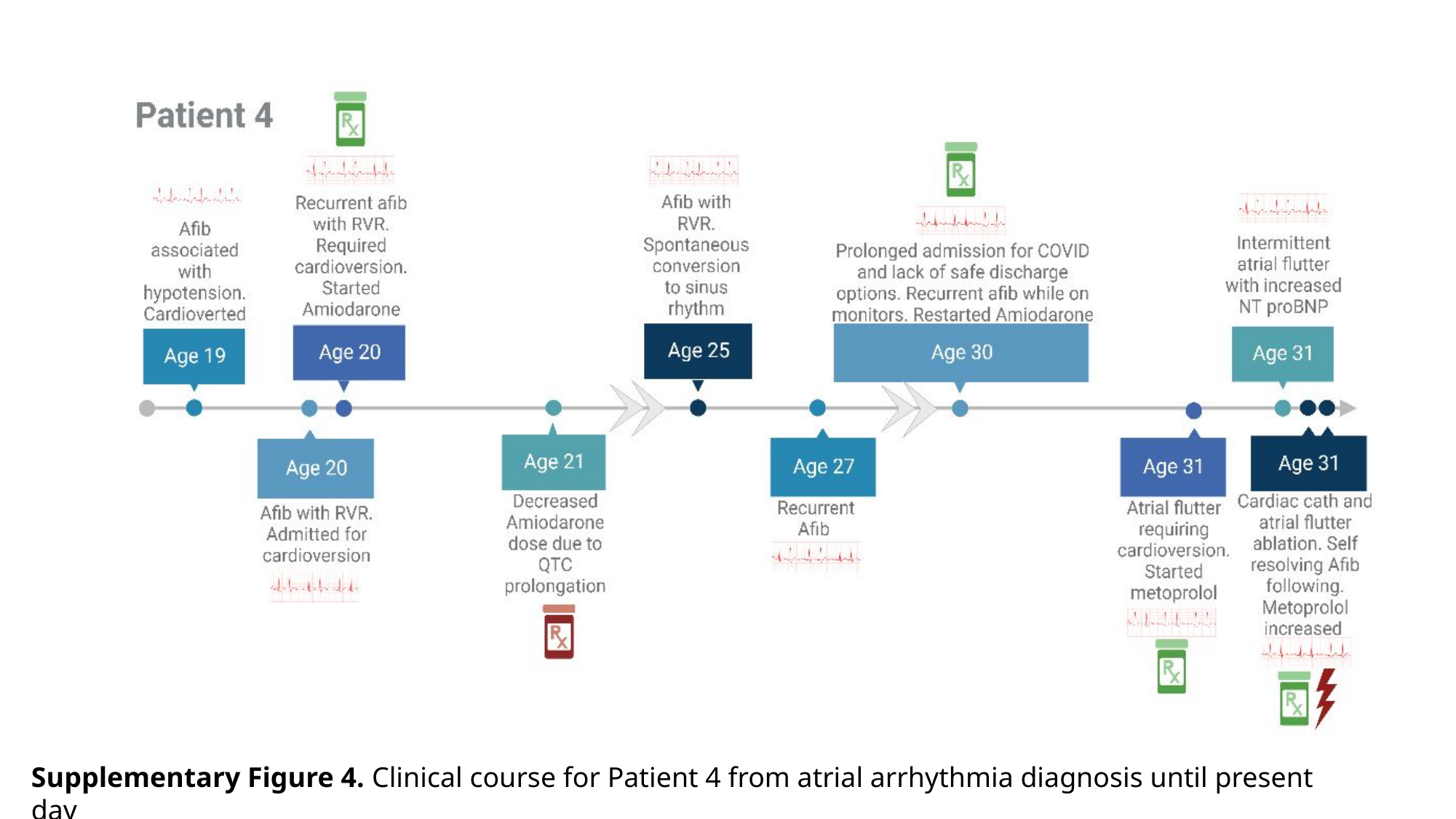

Supplementary Figure 4. Clinical course for Patient 4 from atrial arrhythmia diagnosis until present day

## Slide 5
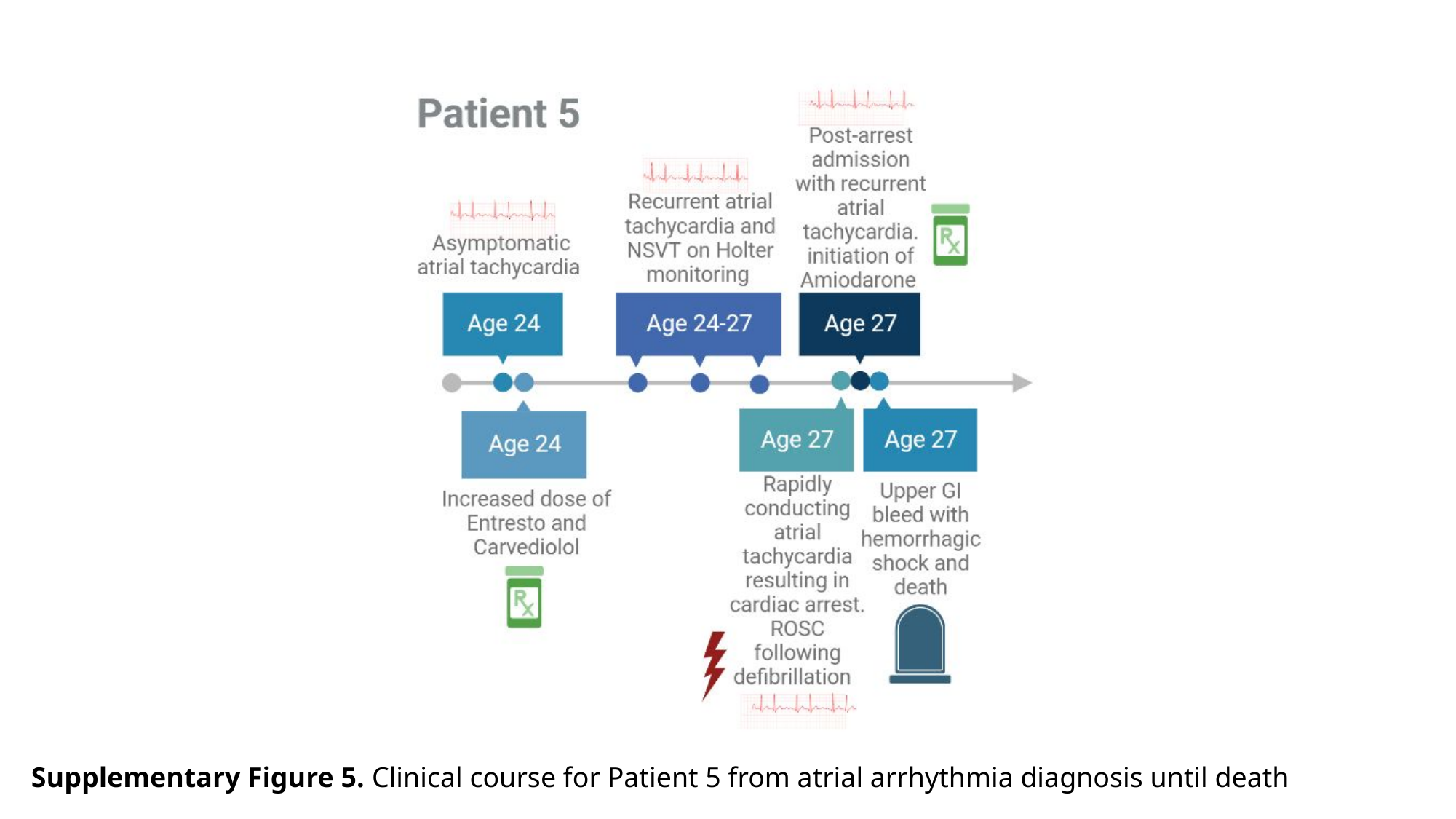

Supplementary Figure 5. Clinical course for Patient 5 from atrial arrhythmia diagnosis until death
